# Shadow Coaching Improves Patient Experience for English-Preferring Patients but not for Spanish-Preferring Patients

**DOI:** 10.1007/s11606-023-08045-2

**Published:** 2023-02-16

**Authors:** Denise D. Quigley, Marc N. Elliott, Mary E. Slaughter, Efrain Talamantes, Ron D. Hays

**Affiliations:** 1 RAND Corporation, 1776 Main Street, Santa Monica, CA 90407 USA; 2 AltaMed, 2040 Camfield Avenue, Los Angeles, CA 90040 USA; 3 UCLA David Geffen School of Medicine & Department of Medicine, 1100 Glendon Avenue, Los Angeles, CA 90024-1736 USA

**Keywords:** coaching, patient experience, CAHPS, language, spline models

## Abstract

**Background:**

Shadow coaching, a type of one-on-one provider counseling by trained peers, is an effective strategy for improving provider behaviors and patient interactions, but its effects on improving patient experience for English- and Spanish-preferring patients is unknown.

**Objective:**

Assess effects of shadow coaching on patient experience for English- and for Spanish-preferring patients.

**Design:**

We analyzed 2012–2019 Clinician and Group Consumer Assessment of Healthcare Providers and Systems (CG-CAHPS) data (*n*=46,089) from an urban Federally Qualified Health Center with 44 primary care practices and 320 providers. One-third (*n*=14,631) were Spanish-preferring patients. We fit mixed-effects regression models with random effects for provider (the level of treatment assignment) and fixed effects for time (a linear spline for time with a knot and “jump” at coaching date), patient characteristics, and site indicators, stratified by preferred language.

**Participants:**

The 74 providers who had a 6-month average top-box score on the CAHPS overall provider rating below 90 (on a 100-point scale) were shadow coached. Similar percentages of English-preferring (45%) and Spanish-preferring patients (43%) were seen by coached providers.

**Intervention:**

Trained providers observed patient care by colleagues and provided suggestions for improvement. Verbal feedback was provided immediately after the observation and the participant received a written report summarizing the comments and recommendations from the coaching session.

**Main Measures:**

CG-CAHPS Visit Survey 2.0 provider communication composite and overall provider rating (0–100 scoring).

**Key Results:**

We found a statistically significant 2-point (small) jump in CAHPS provider communication and overall provider rating among English-preferring patients of coached providers. There was no evidence of a coaching effect on patient experience for Spanish-preferring patients.

**Conclusions:**

Coaching improved care experiences for English-preferring patients but may not have improved patient experience for Spanish-preferring patients. Selection and training of providers to communicate effectively with Spanish-preferring patients is needed to extend the benefits of shadow coaching to Spanish-preferring patients.

## INTRODUCTION

Healthcare organizations strive to improve patient care experience and often focus on providing effective communication between clinicians and patients. This avenue is chosen because good provider communication is crucial to the doctor-patient relationship^1,2^ and a critical aspect of patient experience.^3–6^

Healthcare organizations also increasingly pursue health equity, “striving for equal opportunities for all social groups to be as healthy as possible, with selective focus on improving conditions for those groups who have had fewer opportunities.”^7^ Pursuing health equity necessitates analyzing data to understand differential effects of interventions and improvement efforts. Consumer Assessment of Healthcare Providers and Systems (CAHPS®) patient experience measures, the national standard for collecting, tracking, and benchmarking patient care experiences across settings including ambulatory care,^8–11^ are typically used to monitor and target modifiable provider behaviors to improve patient experience.^12,13^ CAHPS data can also be used to analyze the experiences of different patient subgroups.^14–16^

With the US Hispanic population increasing, and one-third having limited English proficiency, ensuring that providers have effective communication strategies for all their patients is critical. As of 2020, 18.7% (62.1 million) of the US population is Hispanic, an increase of 16.3% from 2010.^17^ The US population is estimated to reach 106 million by 2050.^18^ In addition, the US census indicated that 28% of Hispanics in the US had limited English proficiency, 42% spoke English very well, and 28% spoke only English.^19^ Therefore, incorporating effective communication and interaction strategies to serve the growing Hispanic, Spanish-preferring patient population is crucial for most health care providers. Many health-related settings, including primary care, provide interpreter services for their non-English speaking patients; however, there is mounting evidence that patients have language needs that go beyond simply needing an interpreter for them to receive the best medical care and care experience.^20–22^

Healthcare organizations often use training to change or improve physician behavior and interactions with patients. Some groups and practices use individualized feedback or one-on-one provider counseling, known as “shadow coaching.” This is a type of collaborative learning^23,24^ that uses peers as coaches, who enter into an equal, noncompetitive voluntary relationship with those they coach, observe providers in real-time encounters at a point-of-care, and provide individualized structured, specific feedback to improve task performance and support positive changes.^25–30^ Sessions usually occur in dyads^31^ during a half- or full-day to observe several patient encounters.^32,33^ Mutual trust between recipients and coaches is essential for successful peer-coaching.^34–37^ Traditional medical professional mentorship is a long-term process through which an experienced person (the mentor) guides another (the mentee) in developing skills and knowledge for their professional development.^38^ The aim of mentorship is to enhance the abilities of the mentee, building their capacity to produce the desired career outcomes. Shadow coaching differs from mentorship in that it involves a half-to-full day of observation of direct patient care and provides specific oral and written feedback about how to improve provider-patient interactions; in contrast, mentorship is a long-term relationship with feedback about a large array of topics related to career and professional development.

Additionally, the recommendations from coaches to providers that were supplied via written feedback reports highlight the content and actionability of the coach-provider interchange in shadow coaching.^39^ The authors coded 1082 recommendations found in 92 shadow coaching reports. About half of the recommendations encouraged consistency of existing behaviors and half encouraged new behaviors. Most recommendations related to behaviors of the provider rather than support staff and targeted actions within the exam room rather than other spaces (e.g., waiting room). The most-common recommendations were about behavioral aspects of provider communication and targeted verbal rather than non-verbal communication. In addition, most recommendations were deemed actionable (i.e., specific, descriptive) and encouraged new behaviors rather than encouraged existing actions. Recommendations to providers aimed at improving their interactions with patients need to not only suggest the exact behaviors assessed directly by patient experience surveys but also include actions indirectly associated with those measured behaviors.

Shadow coaching has proven to be effective, with some studies finding that coaching helps build and maintain competencies among physicians, nurses, and other staff, and increases compliance with practice guidelines.^40–42^ Quigley et al. examined patient experience scores before and after coaching that incorporated features consistent with the literature on successful behavior change: a learner-centered approach, immediate feedback, written recommendations on what skills to practice and suggested behavior change.^43^ They found significant immediate improvement in patient experience CAHPS measures of provider communication and overall provider rating following coaching with the gains for coached providers eroding and disappearing after 2.5 years. However, it is not known whether the effectiveness of shadow coaching differs for patients with limited English proficiency and prefer Spanish. We examine whether shadow coaching was similarly effective for Spanish-preferring and English-preferring patients.

## METHODS

### Setting

The study was conducted in a large, urban Federally Qualified Health Center (FQHC) in California that had a quality monitoring system based on the Clinician and Group CAHPS (CG-CAHPS) Visit Survey 2.0 overall provider rating and provider communication composite completed by adult patients or parents of pediatric patients.^9^ Provider communication was selected, as it is the CAHPS composite with the highest correlation with the overall rating of care,^3^ meaning provider communication is the strongest “driver” of a patient’s overall rating.

Shadow coaching was part of the FQHC’s quality monitoring and improving of patient care experiences. Every 6 months, in January and July since 2015, the FQHC calculated every provider’s average 6-month score on the CG-CAHPS overall provider rating (scored with a 0–100 possible range, higher scores are better). The 74 providers with a 6-month average top-box score below 90 in the 6 months prior to calculation were selected for coaching. Details of the coaching intervention and its evaluation are described elsewhere.^43–45^

### Intervention

Eight full-time, high-performing providers (identified on the basis of patient experience and other performance indicators) were selected to shadow other providers for 4 or more patient encounters during a half-to-full day. Coaches attended a one-day coaching seminar by the SullivanLuallin group.^32,46–48^ Provider assignments were based on geography; coaches were assigned regions to minimize their commuting time. Medical director coaches were not permitted to coach providers who reported to them. The shadow coaches observed providers, and, after the observation, provided verbal feedback about strengths and areas of improvement with a focus on patient-provider interactions. Coach feedback was based on their own experiences as high-performing physicians and broader insights into what makes for high-quality patient-provider communication derived from the coaching seminar. This initial feedback was followed by a written coaching report from the coach to the provider summarizing the comments and recommendations from the coaching session.^32^ The primary goal of the shadow coaching session was to identify and target areas of patient-provider interaction that a provider could improve when interacting and caring for their patients, with a focus on provider communication. Coaching occurred from March 2015 to August 2018.

### Data and Analysis

The analytic sample consisted of 46,452 patients who completed the CG-CAHPS Visit Survey 2.0 assessing care from 320 providers from 2012 to 2019; 363 had missing information for provider rating and 12 missing information for provider communication. We compared provider and patient characteristics for the 46,089 respondents with data on both outcomes, who completed the survey in English vs. Spanish (see Table 1 for provider and survey characteristics and Table 2 for patient characteristics) using *t*-tests and chi-square tests.

**Table 1:** Surveys and provider characteristics, overall and by survey language

**Characteristics** **Surveys and providers**	**Overall** ***N*=46,089**	**English language survey** ***N*=31,458**	**Spanish language survey** ***N*=14,631**
	100%	68.3%	31.7%
Number of providers, *N*	320	317	310
Number of coached providers, *N*	74	74	74
Number of Spanish-qualified providers	31	31	31
Number of Spanish-qualified *coached* providers, *N*	12	12	12
Surveys for visits with coached providers, *N* (%)	20,564 (44.6%)	*14,209* *(45.2%)*	*6355* (43.4%)***
Surveys for visits with Spanish-qualified providers, *N* (%)	10,100 (21.9%)	*6084* *(19.3%)*	*4016* (27.4%)***
Surveys for visits with Spanish-qualified *coached* provider, *N* (%)	5200 (25.3%)	*3197* *(22.5%)*	*2003* (31.5%)***

*CG-CAHPS* Consumer Assessment of Healthcare Providers and Systems Clinician and Group Survey, *SD* standard deviation. *Italics* indicates statistically significant differences between survey language groups. *P*-value significance key: *P < 0.05; **P < 0.01; ***P < 0.001

**Table 2:** Patient characteristics, overall and by survey language

**Patient characteristics**	**Overall**	**English language survey**	**Spanish language survey**
Adult, *N* (%)	37,816 (82.0%)	25,853 (82.2%)	11,963 (81.8%)
Age (year)***			
0–17	8,307 (18.0%)	*5,628 (17.9%)*	*2,679 (18.3%)*
18–24	2,919 (6.3%)	*2,717 (8.6%)*	*202 (1.4%)*
25–34	6,589 (14.3%)	*5,919 (18.8%)*	*670 (4.6%)*
35–44	6,248 (13.6%)	*4,582 (14.6%)*	*1,666 (11.4%)*
45–54	8,104 (17.6%)	*5,265 (16.7%)*	*2,839 (19.4%)*
55–64	9,735 (21.1%)	*5,785 (18.4%)*	*3,950 (27.0%)*
65+	4,187 (9.1%)	*1,562 (5.0%)*	*2,625 (17.9%)*
Highest level of education, *N* (%) ***			
<= 8th grade	5,815 (12.6%)	*933 (3.0%)*	*4,882 (33.4%)*
Some high school	5,404 (11.7%)	*2,640 (8.4%)*	*2,764 (18.9%)*
High school grad	9,991 (21.7%)	*7,064 (22.5%)*	*2,927 (20.0%)*
Some college	13,428 (29.1%)	*12,384 (39.4%)*	*1,044 (7.1%)*
4-year coll. grad.	4,607 (10.0%)	*4,265 (13.6%)*	*342 (2.3%)*
4+ years college	3,044 (6.6%)	*2,722 (8.7%)*	*322 (2.2%)*
Missing	3,800 (8.2%)	*1,450 (4.6%)*	*2,350 (16.1%)*
Racial/ethnic group, *N* (%) ***			
Hispanic	31,938 (69.3%)	*18,605 (59.1%)*	*13,333 (91.1%)*
White (non-Hispanic White)	5,821 (12.6%)	*5,742 (18.3%)*	*79 (0.5%)*
Black	768 (1.7%)	*766 (2.4%)*	*2 (0.0%)*
Asian Pacific Islander	3,020 (6.6%)	*3,008 (9.6%)*	*12 (0.1%)*
American Indian/American Native	100 (0.2%)	*94 (0.3%)*	*6 (0.0%)*
Other/multiple races	1,263 (2.7%)	*1,203 (3.8%)*	*60 (0.4%)*
Unknown	3,179 (6.9%)	*2,040 (6.5%)*	*1,139 (7.8%)*

*Italics* indicates statistically significant differences between survey language groups. *P*-value significance key: *P < 0.05; **P < 0.01; ***P < 0.001

Separate mixed-effects linear regression models predicted the CG-CAHPS overall provider rating and provider communication measures. The two models were stratified by language and included a random effect for provider (the level of randomization) and fixed effects for time (represented with a linear spline for time with a knot at coaching date), patient characteristics (age, sex, general health status, education), and indicators for practice. This spline model allows for a change in slope (for a gradual change in scores) and for a “jump” (a vertical discontinuity for instantaneous change in scores) at the date of the intervention and a hypothesized change in scores (the “knot”). Allowing the trajectory to change at the time of coaching independently for the coached and uncoached groups addressed the possible threat of regression to the mean associated with performance-based treatment assignment. Uncoached providers were compared with coached providers by evaluating score changes at coaching and the slope following coaching. These two main effects in the model allow us to assess whether patients of providers who were coached had a significant change or jump in scores immediately following coaching and whether this jump declined after coaching over the remaining study period.

We fit separate models for each of the two language groups corresponding to the stratification variable (i.e., patient preferred language) for both outcomes, for a total of four models. A two-sided, 0.05 significance level was used. All analyses were conducted using the statistical software R and SAS. Study protocols were approved by RAND’s Human Subjects Protection Committee (IRB_Assurance_No: FWA00003425; IRB_Number: IRB00000051).

## RESULTS

### Patient Characteristics

One-third (32%, *n*=14,631) were Spanish-preferring and 68% were English-preferring patients. Forty-three percent of Spanish-preferring patients (*n*=6355) and 45% (*n*=14,209) of English-preferring patients were seen by coached providers. For both languages, 82% were for adult patient visits. Spanish-preferring patients tended to be older, with a mean age of 46 years versus 37 years of age for English-preferring patients. Having a 4-year college degree was higher among English-preferring patients versus Spanish-preferring patients, 22% vs. 5% respectively. Also, as expected, a much higher percentage of Spanish-preferring patients were Hispanic (91% versus 59% for English language surveys). Tests of patient characteristic differences were all significant, except for adult versus child visits, with *p*-values < 0.001. Models adjusted for patient age, sex, general health status, and education and random effect indicators for practice.

### Spanish Qualification of Providers

Thirty-one of 320 providers (10%) were Spanish qualified (i.e., passed assessments in speaking, reading, and communicating fluently in Spanish in a medical setting), including 12 of the 74 coached providers (16%) (see Table 1). Nineteen percent of English-preferring and 27% of Spanish-preferring patients were seen by Spanish-qualified providers; these proportions were 23% and 32%, respectively among patients seen by coached providers.

### Overall Provider Rating

In the preferred-language stratified models, the coefficient for the immediate change at coaching (i.e., jump) for Spanish-preferring patients was non-significant and less than half the magnitude of the jump for English-preferring patients: 0.9 for Spanish-preferring patients with coached providers (95% CI −0.7 to 2.6, *p*-value = 0.28) versus 2.2 for English-preferring patients with coached providers (95% CI 0.7 to 3.8, *p*-value = 0.0037) (see Table 3). The differential change in slope at coaching for English-preferring patients with a coached provider had an estimated decrease of 1 point for every year following coaching (95% CI −1.8 to −0.1, *p*-value = 0.025). For Spanish-preferring patients of coached providers, a decline was not observed.

**Table 3:** Mixed effects linear spline model results, overall and by survey language

		**Stratified models**	
	**Overall** **(Initial study results)+**	**English language survey**	**Spanish language survey**
Overall provider rating	*N*=46,089	*N*=31,458	*N*=14,631
Differential immediate change (i.e., jump) at coaching for coached	*2.0* (CI: 0.8 to 3.2)***	*2.2* (CI: 0.7 to 3.8)**	0.9 (CI: −0.7 to 2.6)
Differential change in slope at coaching for coached	−*0.8* *(CI:* −*1.5 to* −0.1)*	−*1.0* *(CI:* −*1.8 to* −0.1)*	−0.3 (CI: −1.2 to 0.6)
Provider communication	*N=46,440*	*N=31,614*	*N*=14,826
Differential immediate change (i.e., jump) at coaching for coached	*1.9* (CI: 0.7 to 3.2)**	*2.0* (CI: 0.5 to 3.6)*	0.9 (CI: −1.0 to 2.8)
Differential change in slope at coaching for coached	−*0.8* *(CI:* −*1.4 to* −0.05) *	−*0.9* *(CI:* −*1.8 to* −0.01) *	−0.2 (CI: −1.2 to 0.9)

CI indicates confidence intervals. **+** indicates results are from initial study Quigley et al 2021. Models adjusted for patient characteristics (age, sex, general health status, education) and indicator variables for practices. *Italics* indicates statistically significant. *P*-value significance key: *P < 0.05; **P < 0.01; ***P < 0.001

### Provider Communication

For the provider communication composite, the trends are similar. The coefficient for the jump in provider communication for Spanish-preferring patients was non-significant and less than half the magnitude of the jump for English-preferring patients: 0.9 for Spanish-preferring patients with coached providers (95% CI −1.0 to 2.8, *p*-value = 0.34) versus 2.0 for English-preferring patients with coached providers (95% CI 0.5 to 3.6, *p*-value = 0.011) (see Table 3). Also, like the findings for the overall provider rating, the differential change in slope at coaching for English-preferring patients with a coached provider had an estimated decline in score of −0.9 every year following coaching (95% CI −1.8 to −0.01, *p*-value = 0.047). For Spanish-preferring patients of coached providers, a decline was not observed.

## DISCUSSION

Shadow coaching has been previously shown to improve the CG-CAHPS Visit Survey 2.0 overall provider rating and provider communication scores.^43^ This study examines whether these improvements differ by patient language preference in a FQHC primary care setting with similar proportions of English-preferring and Spanish-preferring patients. We found significant improvements in both the overall provider rating and provider communication composite from coaching for English-preferring patients and no clear evidence of such gains for Spanish-preferring patients. Other studies show that even within racial and ethnic group, mean reported experiences for non-English-preferring patients are worse than English-preferring patients,^16,49^ highlighting the need to provide excellent patient experience to all language groups. Taken together, these findings suggest additional actions at the provider and patient level and at the organizational level to improve the effectiveness of shadow coaching for Spanish-preferring patients during direct patient care to ensure that Spanish-preferring patients receive linguistically appropriate care and effectively navigate the health system. For example, coaches can observe and provide feedback to assist non-other language fluent providers know when to call an interpreter^50^ and have an accurate gauge of their own limitations.^51^

The apparent difference in coaching effect for English- and Spanish-preferring patients suggests that the content of current coaching protocols should be revisited and refined with the input of providers who are especially effective with Spanish-preferring patients. Notably, only one-third of Spanish-preferring patients in the coached provider group of the study were seen by a Spanish-qualified coached provider. Coaching for providers with significant Spanish-preferring patient volume could also be arranged to include observation of interactions with both English-preferring and Spanish-preferring patients to ensure that the coach is able to recommend improvements unique to care for Spanish-preferring patients. Revision of protocols might include examination of and improvement in the provision of patient materials (e.g., education materials, visit summary instructions, medication information), linguistic support (e.g., bilingual qualified providers, medical interpreters, or translators available)^52,53^, and other areas of cultural competencies (e.g., including family members in medical discussions, working with an extended care team) for Spanish-preferring patients.

In addition, these findings also highlight the need to investigate the potential impact of provider-patient language concordance on patient experience and how language concordance impacts patient-provider interactions. Specifically for the shadow coaching program, selection and training of providers caring for Spanish-preferring patients could be refined to extend benefits of shadow coaching to Spanish-preferring patients. In particular, it may be challenging for providers to fully confer the lessons of shadow coaching unless they have mastery of a patient’s preferred language.

Our study has limitations. First, this work is based on a single health care organization that is a large FQHC, so our findings may not be generalizable to other medical care settings. Additionally, two providers who were coached were not included in the analysis because of missing patient experience surveys either before or after their coaching data. Also, coached providers might unrepresentatively present only their best behaviors when being observed but observing a provider for a half to full day should still allow sufficient patient interactions for the coach to provide input and feedback on how a provider can improve their care for patients. Lastly, we defined Spanish-preferring patients based on the survey language of the completed CG-CAHPS survey, a proxy of actual language preference. Finally, the confidence intervals of treatment effects for English-preferring and Spanish-preferring patients overlap, so while there is clear evidence of a treatment effect for English-preferring patients and no such evidence for Spanish-preferring patients, there is not statistically significant evidence that the treatment effect differs by language preference (i.e., an interaction of treatment with language preference.

## CONCLUSION

Health care organization dually strive to improve patient care experience and health equity. Patient-provider interactions can be improved through one-on-one provider counseling that includes patient-care observation and individualized recommendations from trained peers, known as shadow coaching. Such coaching improved care experiences, as measured by the CAHPS overall provider rating and provider communication composite, overall and for English-preferring patients but may not have improved Spanish-preferring patient experiences. Targeted refinement to shadow-coaching, targeted coaching of language-concordant providers, and direct assistance of Spanish-preferring patients may broaden the benefits of shadowing and increase its contributions to improving health equity.
